# Analyzing the Combined Effect of Multiple Environmental Factors on Fish Distribution, by Means of the Mixed Distribution–Decomposition Approach, as Illustrated by the East China Sea Hairtail

**DOI:** 10.3390/biology12071009

**Published:** 2023-07-15

**Authors:** Yong Liu, Jia-Hua Cheng

**Affiliations:** Key Laboratory of East China Sea Fishery Resources Exploitation and Utilization, Ministry of Agriculture and Rural Affairs, East China Sea Fisheries Research Institute, Chinese Academy of Fishery Sciences, Shanghai 200090, China; liuy@ecsf.ac.cn

**Keywords:** East China Sea, hairtail, environmental factors, distribution

## Abstract

**Simple Summary:**

Understanding the factors that influence the behavior and distribution of creatures is essential for studying the interaction between environment and biology. In this study, we focused on the main environmental factor, water temperature, and its impact on the distribution of hairtail, a specific species. By examining the spatial limits of the hairtail’s temperature range, we found that temperature alone does not fully explain its distribution. We expanded our investigation to include salinity and water depth as additional factors. The results showed that the combination of multiple factors significantly reduced the intersection range between hairtail and the defined environmental factors. This suggests that each factor contributes differently to hairtail distribution. To further explore differences in organism aggregation, future research might compare aggregated and non-aggregated variables related to specific areas of water in terms of specific factors. Moreover, we observed that hairtail distribution varied seasonally, with different levels of coverage and proximity to shore. These findings emphasize the complexity of the organism’s habitats and the need for a comprehensive understanding of environmental influences.

**Abstract:**

An organism’s habits are formed primarily as a result of environmental circumstances. Analyzing an organism’s habits and examining their causes requires a thorough understanding of the peculiarities of an organism’s living environments. Analyzing the environmental factors necessary for an organism’s survival is a crucial component of studying how the environment and biology interact. The favorable temperature range for the species has been discovered in previous investigations of the hairtails’ main water temperature distribution range, covering both the water regions with and without the hairtails. It is implied that there may be other elements besides water temperature that also affect dispersion. The hairtail, though, is still the subject of the study. To investigate and confirm the corollaries, salinity and water depth were added as variables. We observed that the intersection of the main ranges of two environmental factors, as well as the main hairtail range of interest, were greatly reduced when compared to a single factor range; the sum of the three factors will further increase the reduction. The primary cause is that the main range of hairtail relative to each factor is incomplete, and the target bodies specified by various factors are very varied. To further investigate the factors that affect the distribution of the organism’s active areas, a comparison between aggregated and non-aggregated waters relative to one factor can be done in the next step. A good sequence of environmental elements, namely temperature>salinity>water depth, is obtained in the above analysis procedure by comparing the accuracy and significance of each factor for the primary range of the hairtail. Additionally, it was noted that the main population of the hairtail covered different areas depending on the season, with less coverage in spring and autumn and greater coverage in summer and winter. The main part of the hairtail population also tended to be distributed closer to the coast in summer and winter, and farther offshore in spring and autumn. These seasonal variations may be related to the two distinct reproductive cycles of hairtail, occuring in spring and autumn.

## 1. Introduction

Since adaptation to the environment is necessary for an organism’s survival, environmental forces are the primary driver of the development of an organism’s habits. The Earth’s continents have various landforms and are affected by various environmental factors, creating a great diversity of habitats that support a vast diversity of individual creatures and biological communities. Understanding the behavior of living beings, observing their systems, and understanding the characteristics of their environment are fundamental and necessary processes. There is still much room for in-depth research into the relationship between marine organisms and the marine environment due to the complex environment of the ocean, which has given rise to a wide variety of marine organisms and their unique habits. Due to the unique nature of the marine environment, human understanding is not deep enough.

Temperature is a crucial environmental parameter in the marine ecosystem. The growth, development and reproduction of marine creatures at various stages of life are influenced by seawater temperature. The study of the relationships between fisheries, fishing season and water temperature has been conducted from two perspectives: on the one hand, it focuses on the fundamental application of the industry, including primarily the relationship between these three factors [1,2]; on the other hand, it focuses on fundamental theoretical research on the behavior of fishery organisms or fisheries, mainly aiming to analyze the law from a scientific point of view and explore these questions [3,4].

Investigating the environmental conditions necessary for survival is a crucial and fundamental aspect in studying the interactions between the environment and fishery species. Early research was based on a direct description of the minimum and maximum temperature ranges in which fish populations occur [5,6], as well as the temperature range in which the frequency of their occurrence is dominant [7]. A method to analyze the range of water temperatures in terms of the distribution of organisms, in combination with the biomass size of target objects, was recently developed [8]. Based on this method, we further optimized the spatial distribution range of organisms to connect the spatial range of environmental factors and analyze the ranges of environmental factors suitable for organisms intuitively and quickly [9].

During the investigation of the temperature range of the hairtail’s primary distribution, it was observed that the inferred area based on the main environmental range of the hairtail significantly exceeded the actual distribution area [9]. This raises the question of why other areas with similar temperature conditions did not exhibit hairtail aggregations. This implies that temperature alone is not the sole determinant of the hairtail’s distribution. Are there significant environmental elements that restrict hairtail dispersion other than water temperature? To study and address the above questions, we continued to use the hairtail as a research object and added the environmental variables of salinity and water depth, in addition to water temperature.

Hairtail is a species which is representative of bottom-dwelling, traditionally economically significant fish in China. Throughout history, along with the large yellow croaker, the small yellow croaker and the cuttlefish, it has been hailed as one of China’s top four marine products, possessing considerable economic value [10,11]. Hairtail is mainly distributed within the western Pacific and the Indian Ocean, and can be found in several waters around China, including the Yellow Sea, East China Sea, Bohai Sea and South China Sea [12]. In recent years, despite the continuous decline of lower-layer fish resources, the catch volume of upper-layer fish species has increased, and the catch of hairtail is still among the top five in China [13]. Hairtail has a wide distribution range, extensive migratory routes, and a long spawning period [14]. It occupies a significant position in terms of economic and ecological value [15,16]. This study has as its research subject the hairtail. First, the main ranges of temperature, salinity and water depth in which the hairtail was distributed were analyzed. Then, the spatial distribution area of the hairtail was inferred based on these range intervals. Subsequently, we integrated the inferred spatial areas of these three environmental factors to obtain the shared spatial area under the constraints of all environmental factors. Finally, we compared the shared spatial area with the hairtail’s actual distribution area, analyzing whether the accuracy of inferring the spatial area under multiple environmental constraints had improved compared to the single-factor analysis, in order to validate the hypothesis proposed in the previous section.

## 2. Materials and Methods

### 2.1. Material Sources

**Figure 1:**
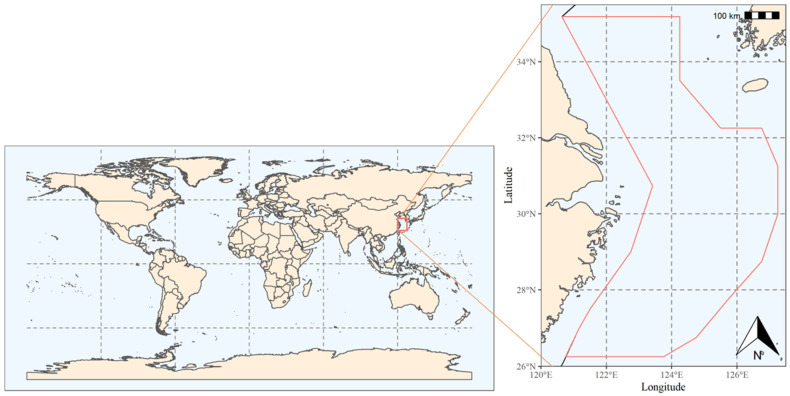
Schematic diagram of the study area: A line along the coast is a fishing ban line, and the area enclosed by the red line is the water area surveyed in this study.

Data were collected in May (spring), August (summer), November (autumn) and January 2015 (winter) from four large-area fixed-station surveys of fisheries resources in the East China Sea area (Figure 1, in the red box). The survey boat was a double trawler with net gear measuring 100 meshes of 4 m, a mesh size of 2.5 cm and an average towing speed of 2 miles per hour. A station was placed at each 30′ interval in longitude and latitude, and the survey region ranged from 26°00′ to 35°00′ N, and from 120°00′ to 127°00′ E. The stations were evenly distributed in a grid pattern. Fishing nets were collected at each station, and the CTD was then lowered to the bottom, raised, and collected after a brief interval in order to collect hydrological data, including water temperature, salinity, and depth. Considering that the surveyed fishing vessels are bottom trawlers that target organisms in the bottom layer of seawater, the environmental factors that influence their distribution should be related to the hydrological conditions of the bottom layer. Therefore, the hydrological data studied in this paper are all from the bottom layer.

### 2.2. Methods

#### 2.2.1. Mixed Distribution–Decomposition Method

When the characteristics of the environmental factor distribution for the main group of the hairtail’s population were examined, it was observed that the hairtail’s main groupings for some seasons showed more than one clearly unique distribution peak. The mixed-density decomposition approach was employed [17] to examine key elements of the various factor distribution peaks, such as the factor value at the distribution’s center, the factor range, the percentage of group number and so forth. The approach assumes that a mixed distribution is simply a proportional combination of different unique distributions, one represented mathematically by a mixed probability density function, or *g*, which is simply the weighted sum of the densities of the *k* components.

$$g\left(x|\pi ,\mu ,\sigma \right)=\pi_{1}f\left(x|\mu_{1},\sigma_{1}\right)+\ldots +\pi_{k}f\left(x|\mu_{k},\sigma_{k}\right)$$

Component densities can be normal, logarithmic, gamma, exponential, Weibull, or other distribution-type densities. Variables include the mean and standard deviation of each component, as well as the mixing ratio. The normal distribution density is used in this paper. Using the mix function from the mixdist function package (0.5–5) [18] in the R language program [19], the specific computation procedure was implemented.

#### 2.2.2. Interpolation Method for Contour Surfaces

In this study, an unbiased optimal estimation of variables in a finite region was performed using the ordinary Kriging interpolation approach, which is based on semi-variance function theory analysis [20]. This approach works best when simulating local trends. The geoR toolbox (1.8-1) [21] of the R statistical software (4.0.1) was used to implement the ordinary Kriging interpolation method.

#### 2.2.3. Calculation Method for Relevant Parameters

Three variables—temperature, salinity and depth—were introduced for investigation, with the aim of analyzing the environmental factors that influence the spatial distribution of the hairtail. To assess the impact of each factor on the distribution, this study calculated an effective area ratio (*R_effective_*) by comparing the intersection area (effective area, *A_effective_*) of the environmental factor’s main range (*A_env_*) and the organism’s main range (*A_bio_*) with *A_env_*. *R_effective_* indicates how well this factor captures the distribution range of the organism. This study also computed a coverage ratio of the organism’s main range, as factor (*R_covering_*), by comparing *A_effective_* with the area encompassed by the organism’s main range. *R_covering_* reflects how important the factor is for describing the organism’s range. The calculation equation that applies is as follows:

$$R_{effective}=\frac{A_{effective}}{A_{env}}=\frac{A_{env}\cap A_{bio}}{A_{env}}$$

$$R_{covering}=\frac{A_{effective}}{A_{bio}}=\frac{A_{env}\cap A_{bio}}{A_{bio}}$$

It is quite challenging to determine the regions of the studied objects, because their distribution ranges are all irregular polygons; therefore, an approximative approach was employed in this work. First, a reference array of points was created in the search region, one consisting of a dense array of evenly spaced points. The area was then translated into a point count, because the number of points is proportional to the size of the study area. When determining the overlapping area, the measurement was converted to determine the number of points shared by the two areas; when determining the overlapping area with multiple factors, the number of points shared by multiple areas was determined.

### 2.3. Softwares Used

The R language program (4.0.1) [20] was used to implement all data processing and graphics in this paper. RODBC (1.3-17) [22] and XLConnect (1.0.5) [23] were the primary function packages used to process and organize data. Mapdata (2.3.0) [24], maps (3.3.0) [25], mapplots (1.5.1) [26] and ggplot2 (3.3.6) [27] were the primary function packages used for painting.

## 3. Results

### 3.1. The Major Range of the Resource’s Distribution

**Figure 2:**
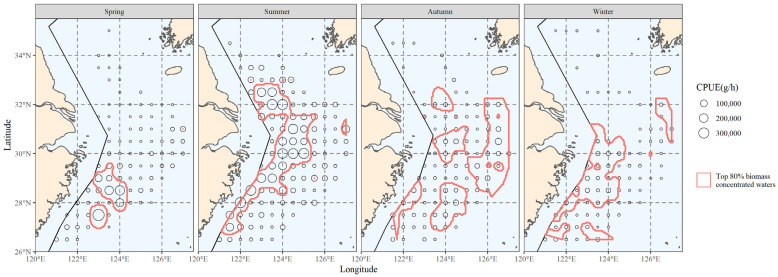
The spatial distribution of the top 80% of hairtail density levels at various seasons.

The top 80% of biomass in the hairtail’s locations was scattered within the areas delimited by the red line in Figure 2. These waters serve as the primary habitat for hairtail throughout the year, the primary fishing grounds for fish production and the main habitat for hairtail, as determined in this study. This range served as the primary distribution for the investigation of the main distribution range of environmental components.

### 3.2. Temperature Distribution Analysis

#### 3.2.1. Temperature Dispersion of the Major Hairtail Population Areas

**Figure 3:**
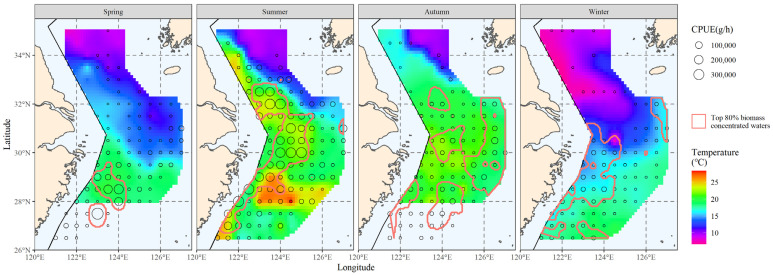
The correlation between bottom water temperature and the spatial distribution of the top 80% of hairtail density levels at various seasons.

The bottom-water temperature distribution for each season was calculated by interpolating the data from the survey at each station. The hairtail’s principal distribution range from the previous section was then superimposed on the water temperature distribution results to produce the initial data for the subsequent principal temperature study (Figure 3).

#### 3.2.2. The Temperature Range Corresponding to the Major Hairtail Population Areas

**Figure 4:**
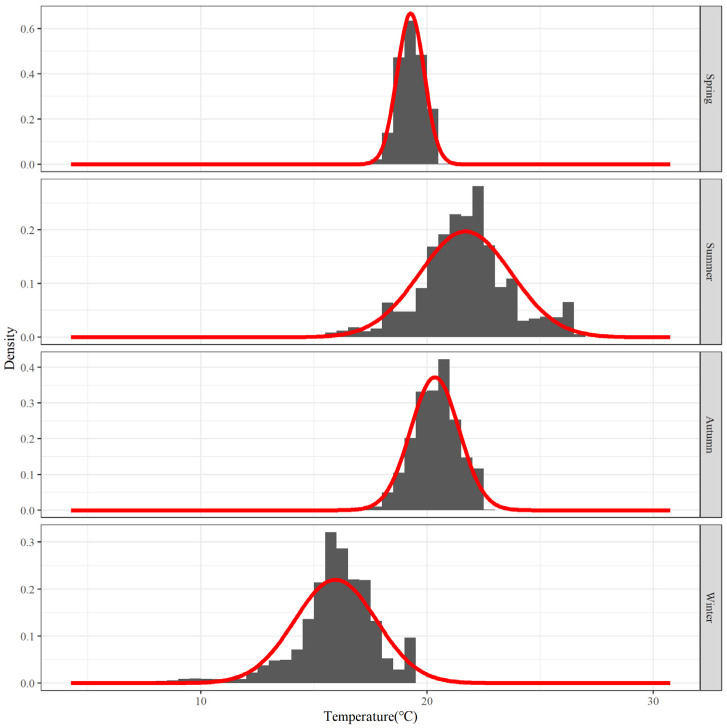
Frequency distribution of water temperature for the top 80% of hairtail density levels at various seasons: The red line is the cumulative distribution of all fitted probability distributions, same below.

Figure 4 shows the frequency distribution of water temperature for each season. There is a very evident aggregation interval in the water temperature each season, and the data distribution pattern is very similar to that of the normal distribution.

The primary water-temperature distribution parameters for each season were derived by decomposing the mixed distribution, and the results of the analysis are presented in Table 1. The average distribution of water temperature is the highest and has the highest distribution of water temperature variation values in the summer. The average water temperature in the autumn and spring is also relatively high, but their variation values are relatively low. In the winter, the average distribution of water temperature is at its lowest and it has a relatively high variation value.

#### 3.2.3. The Area Corresponding to the Main Temperature Range

**Figure 5:**
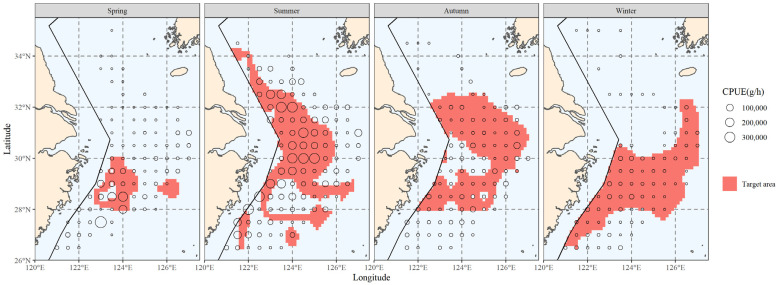
The spatial area corresponding to the primary temperature range of hairtail in various seasons.

The interpolation’s results were used to determine the spatial distribution range of the principal water temperature for the hairtail; they are represented in Figure 5, according to the results in the previous section (see Table 1). When we compare the main water temperature range with the main range of the hairtail (Figure 2), we observe that the former encompasses a wider range of non-target waters, in addition to the main hairtail’s range. These waters have the same ideal water temperature, but there is no hairtail aggregation. This is the starting point of this research, and measurements of increasing salinity and analysis of water depth conditions will be used to further investigate the possibilities.

### 3.3. Salinity Distribution Analysis

#### 3.3.1. Salinity Dispersion of the Major Hairtail Population Areas

**Figure 6:**
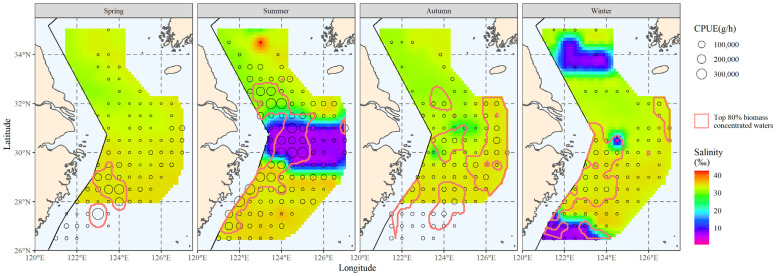
The correlation between bottom-water salinity and the spatial distribution of the top 80% of hairtail density levels at various seasons.

Figure 6 was created by superimposing the distribution range of the main hairtail population over the salinity distribution results. The original data for the main salinity range study in the figure were the salinity data within the main hairtail population’s distribution range.

#### 3.3.2. The Salinity Range Corresponding to the Major Hairtail Population Areas

**Figure 7:**
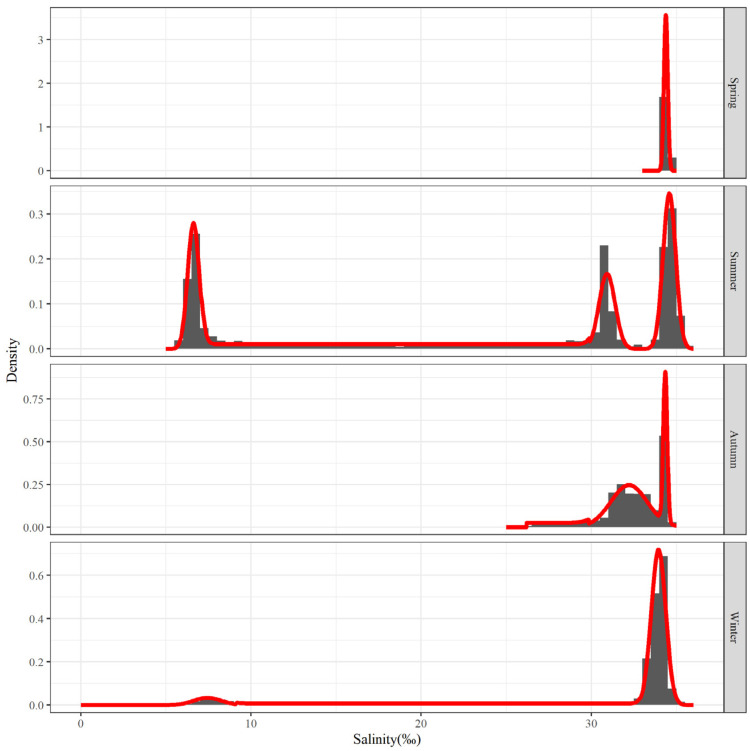
Salinity frequency distribution for the top 80% of hairtail density levels at various seasons.

Characteristics of the main salinity distribution for each season were examined using the salinity data that is displayed above. Figure 7 represents the main salinity frequency pattern for each season. The salinity distribution in summer has three waves that are more clustered together; the clustered waves in autumn and winter consist of two waves, but the two in autumn are more closely spaced, while the two in winter have a larger span; the main salinity distribution during the spring is relatively concentrated, with only one wave.

The salinity of normal seawater is generally greater than 30‰. In summer, there is a large area of water near the Yangtze River estuary with a salinity of less than 10‰. In winter, there are also two small areas of water with a salinity of less than 10‰ in the north and south. The direct cause of these low-salinity waters may be freshwater from the land rushing into the sea. The low-salinity water in summer is caused by the dilution of the water by China’s largest river, as it enters the sea—the Yangtze River [28]. The low-salinity water in the north in winter may be caused by dilution from one of the seven major rivers in China—the Huai River [29]. The low-salinity water in the south may be caused by dilution from the largest river in eastern Fujian—the Jiaoqi River [30].

Table 2 presents the analysis’ results. Except for two peaks of salinity below 10‰ (one in summer and another in winter), the average salinity values of the remaining peaks all remain at a high level, exceeding 30‰, as can be seen in the salinity distribution of the four seasons of the year, which has a high average salinity value of about 34‰. Apart from a variation value above 1 in autumn and a variation value greater than 0.5 in winter, the variation values of the other wave peaks are all below 0.5. Each of the waves of the main salinity distribution has very small variation values.

#### 3.3.3. The Area Corresponding to the Main Salinity Range

**Figure 8:**
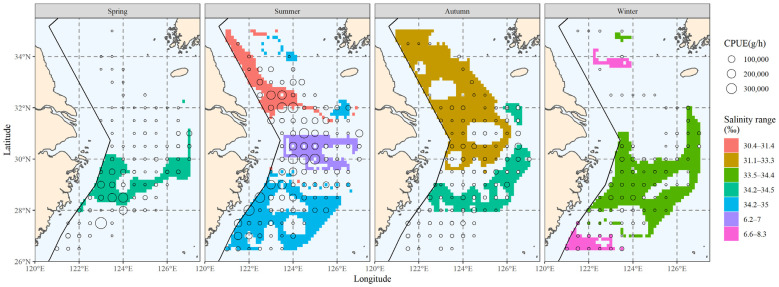
The spatial area corresponding to the primary salinity range of hairtail at various seasons.

The main spatial distribution range of salinity is presented in Figure 8. Comparing the hairtail’s main distribution range (Figure 3), we observe that it differs from the biomass range in that it contains more non-target waters, in addition to partially covering the biomass waters. When compared to the main water temperature range (Figure 5), the main salinity range covers the biomass waters relatively less effectively. Additionally, outside of spring, the salinity range had insufficient spread to cover biomass waters. Apart from spring, the salinity range also showed greater fragmentation and could not be linked to a single large contiguous area of water.

### 3.4. Depth Distribution Analysis

#### 3.4.1. Depth Dispersion of the Major Hairtail Population Areas

**Figure 9:**
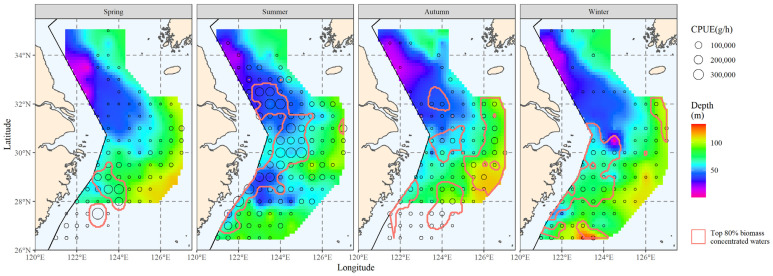
Correlation between water depth and the spatial distribution of the top 80% of hairtail density levels at various seasons.

The depth distribution for each season was calculated, and the distribution range of the major hairtail population was then superimposed on the results. The main depth data were obtained within the main hairtail population’s distribution range in Figure 9, and these new data served as the bases for further analysis.

#### 3.4.2. The Depth Range Corresponding to the Major Hairtail Population Areas

**Figure 10:**
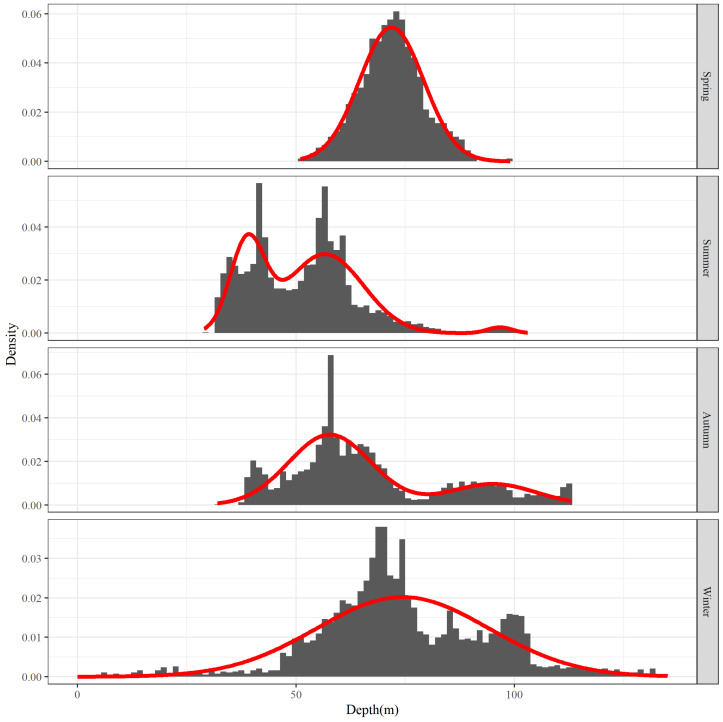
Water depth frequency distribution for the top 80% of hairtail density levels at various seasons.

The main distribution of depth levels for each season was examined. Figure 10 displays the depth frequency distribution for each season. The distribution of the main depth for each season varies slightly. For example, the depth distribution in spring is the most concentrated, while the depth distribution in winter is the most extensive. However, both distributions are grouped by analysis as only one wave. The main depth distribution range in summer and autumn is comparatively low centered, and both have several relatively concentrated wave peaks.

The parameters of the main depth distribution of each season are presented in Table 3. As can be observed, the depth distribution is more concentrated in spring and more widely scattered in winter, but their average depths—both over 70 m deep—are quite close to each other. Furthermore, the characteristics of depth distribution in summer and autumn are more comparable, with both showing multiple peaks; there are three peaks in the summer, along with one of the shallowest levels of aggregated waters of all the seasons, with an average depth of 40 m, which is considered shallow; the average depth of the other two peaks in summer is more compatible with that of the two in autumn, with average depths greater than 50 m and greater than 90 m, respectively.

#### 3.4.3. The Area Corresponding to the Major Depth Range

**Figure 11:**
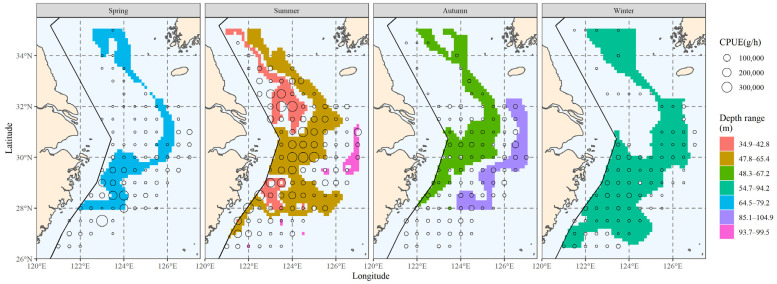
The spatial area corresponding to the primary water depth range of hairtail in various seasons.

After finding the distribution range of the major water depth for hairtail, the relevant results were obtained; they are shown in Figure 11. When we compare the main depth range with the biomass range (Figure 6), we observe that in addition to encompassing a portion of the biomass waters, the main depth range comprises additional non-biomass-distribution waters. The range of the main population’s water depth is more continuous and of a wider expanse without fragmentation than is the main population’s salinity range (Figure 8).

### 3.5. Thorough Examination of Three Environmental Elements

#### 3.5.1. Comparison of Single Factors

**Figure 12:**
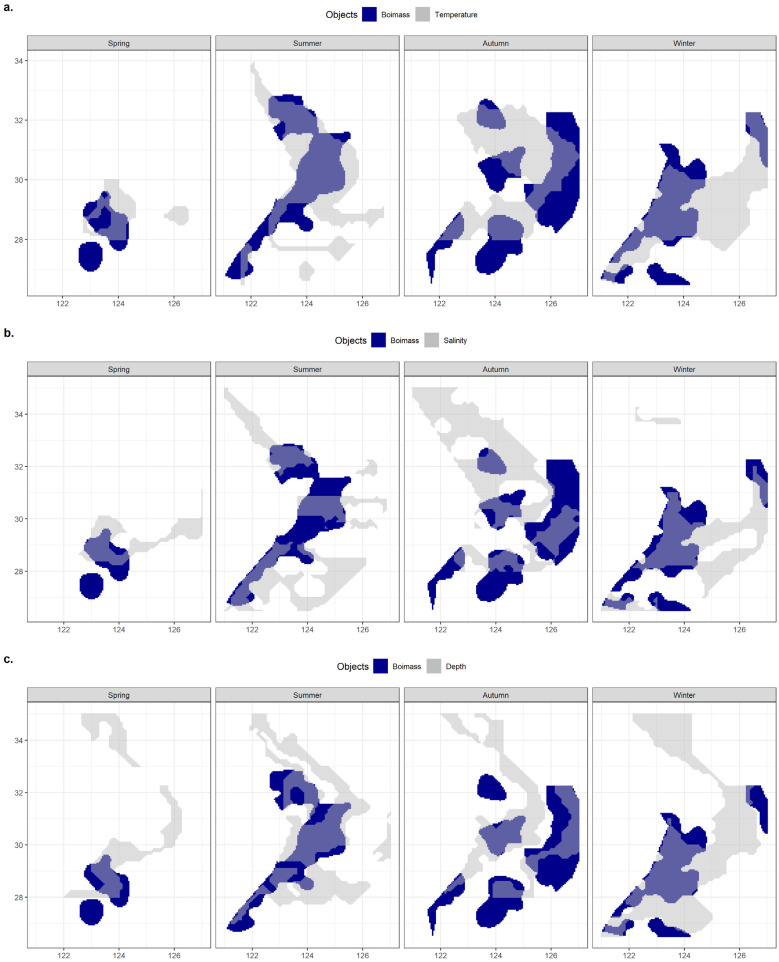
The overlaps of spatial ranges between hairtail populations and environmental factors: (**a**) temperature; (**b**) salinity; and (**c**) depth.

Figure 12 compares the major ranges of the three environmental factors with overlapping biomass ranges to illustrate the advantages and disadvantages of the three environmental factors in reflecting or predicting the hairtail’s spatial range.

Table 4 includes statistical analysis of the effective area, effective ratio and coverage ratio of the overlap between the main range relative to each environmental factor and the main range of the organism in each of the four seasons. Except in winter, where the effectiveness of salinity was higher and the effectiveness of temperature closer to the former, the effectiveness of temperature was the highest among the three environmental factors. Seasonal variations in the effectiveness of salinity in relation to depth can be observed, with salinity effectiveness being greater than that of depth in spring and winter, the opposite in autumn, and similar in summer. The temperature coverage ratio was highest in all seasons except spring, when it was lowest. Salinity coverage was consistently lower than that of depth, except in spring when it was slightly higher.

#### 3.5.2. Comparison of Multi-Factor Analyses

**Figure 13:**
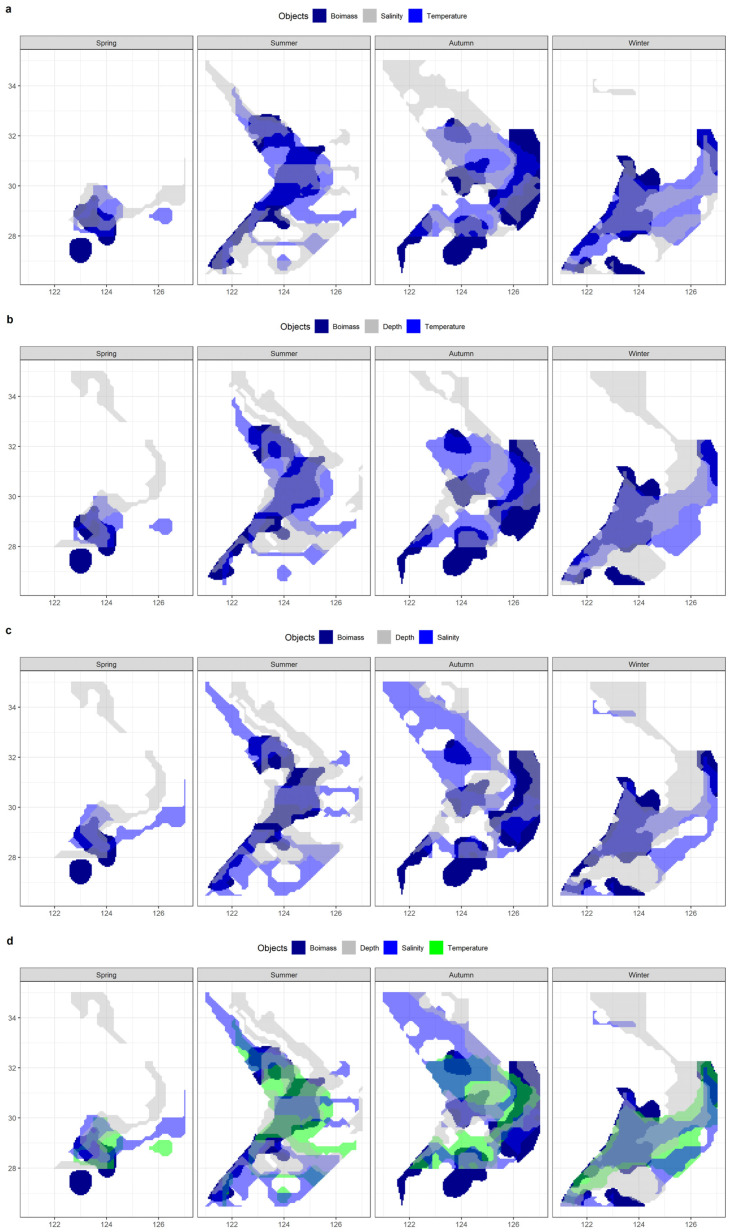
The overlaps of spatial ranges between hairtail populations and environmental multi-factor analyses: (**a**) temperature ∩ salinity; (**b**) temperature ∩ depth; (**c**) salinity ∩ depth; and (**d**) temperature ∩ salinity ∩ depth.

This section integrates observations of the spatial ranges of various environmental factors to compare their effects on biomass range coverage. The overlap between the shared major range of two environmental factors, as well as the overlap between the shared major range of all variables and the major biomass range, are schematically represented in Figure 13.

Table 5 lists the effective areas and the coverage ratio of the overlap between the range of each combination of environmental factors and the main biomass range for the four seasons. Any multi-factor combination has a smaller effective area than any single factor when compared to Table 4. Compared to any two-factor combination, the three-factor combinations have less coverage. The temperature and depth combination had the greatest coverage in summer and winter out of the three two-factor combinations, and the salinity and depth combination had the highest coverage in spring, while the autumn coverage of the three groups was approximately similar and provided the greatest coverage by the combination of temperature and salinity.

## 4. Discussion

### 4.1. The Importance of Multiple Environmental Factors in Improving Hairtail Spatial Range Location Accuracy

The original goal of this study was to increase the number of environmental factors to decrease the comparatively vast spatial extent dictated by a single environmental factor to acquire a significantly more accurate range for displaying the main spatial distribution of the target species. The overlapping area for all dual environmental factors (Table 5) was smaller than that of any individual environmental component (Table 4), and the three-factor composite coverage was smaller than that of the two-factor coverage, meaning that the study’s findings do not match its expectations (Table 5).

The observation that the intersection part of two sets is, in most situations, smaller than either set is not very complicated to understand. However, in the unusual scenario where one set encompasses the other, the intersecting set is occasionally equal to one of the sets. The findings of this study demonstrated that the intersection of the ranges of both environmental factors was significantly reduced when compared to one. Furthermore, the intersection of each environmental factor with the main hairtail population resulted in a similar significant reduction. The multiple range reductions were ultimately reflected in the reduced coverage results after compounding the environmental factors. The coverage, for example, was 22% for the temperature–salinity combination, compared to 39% for temperature alone and 45% for salinity alone, drops of 44% and 51%, respectively. Table 6 presents the coverage decline for each environmental element following various combinations. There were large variations in the coverage of the biomass range by different environmental factors, but the analyses of overlapping environmental factors all led to a notable decline in the intersection of the ranges of the multiple environmental factors with that of the actual biomass. The average single-factor drop of the two-factor composite was classified as (temperature-salinity) > (salinity-depth) > (temperature-depth). In the three-factor composite scenario, the order of the relative size of the drop of the two variables was (temperature–depth) > (salinity–depth) > (temperature–salinity). The two orderings above are completely at odds with each other, but express the same results. For instance, the results clearly stated in the first ordering are the largest differences between the major spatial variation in salinity and temperature, and therefore the greatest reduction after compounding; in the second ordering, when the first combination of temperature and depth is added to the salinity factor, it is precisely because of the enormous difference within temperature that it results in a more substantial reduction in coverage after the combination of the three factors.

By intersecting the ranges of other factors with the range of a previous factor, the study aimed to further improve the accuracy of the range as defined by the single environmental factor, while filtering out part of the non-target range of the previous factor. The empirical findings demonstrate that when constructing the intersection set, some target ranges are also eliminated along with the non-target ranges. This is primarily because the target ranges of the intersection set are actually reduced as a result of the incomplete coverage of the target subjects by the new main population ranges within the environmental factor.

Observing the analysis of the main population range within each environmental factor by frequency, we observe that the number of relatively concentrated target groups of the identified main ranges varied between different seasons and environmental factors, but without a consistent pattern. In summer, for instance, one peak group was confirmed based on temperature frequency (Figure 4), three peak groups were confirmed based on salinity (Figure 7), and three peak groups were confirmed based on water depth (Figure 10); however, despite the same number of groups identified by salinity and water depth, the proportion of each group was relatively different. It can be observed that the groups corresponding to the main population ranges as determined by each environmental factor are distinct. This inconsistency among the groups corresponding to different environmental factors inevitably leads to variations in the main ranges and consequently results in a substantial reduction in the shared target range under multiple-factor combinations.

### 4.2. Comparing the Impact of Different Environmental Conditions on the Hairtail’s Main Spatial Range

In this study, an effective ratio ($R_{effective}$) and a coverage ratio ($R_{covering}$) were used as comparison metrics. The effective ratio, or how much of the environmental spatial range is made up of the hairtail range, indicates how accurately the environmental factor describes the hairtail distribution range. The more accurate the hairtail detection is based on this environmental range, the higher this ratio will be. The results of the study (Table 4) revealed that the environmental factors chosen in this paper did not reliably identify the main spatial range of hairtail, with the highest effective proportion being only about half, meaning that half of the range determined by the environmental factors was not within the distribution range of the main biomass of hairtail. The temperature accuracy was generally the best of the three environmental factors chosen for this study, particularly in spring and summer, and reached its highest accuracy (52% effective rate) in summer. In the other two seasons, it was comparable and the second-best, although not the best. Salinity and water depth performed best in winter and autumn, with salinity having the highest accuracy in the first season (44% effective rate) and water depth in the second (44% effective rate). The main hairtail distribution was accurately predicted by environmental parameters based on temperature, salinity and depth, in decreasing order. It is important to note that while water depth accuracy was higher than salinity accuracy in summer and autumn, water depth accuracy in spring and winter was particularly poor and fluctuating, producing a final average worse than that of salinity, while salinity accuracy and temperature accuracy were relatively stable.

The coverage ratio measures the proportion of the hairtail range within the primary range of the environmental factor to the total hairtail range, which reflects the ability and significance of that environmental factor to locate the targeted range. The results of the study (Table 4) revealed that temperature was the most significant of the three environmental elements and that it was most affected throughout the year, except for spring, when it performed worse. The next factor was water depth, which in all seasons—except spring, when it was less effective than salinity—had coverage greater than salinity. Apart from spring, when it performed relatively better, and the other seasons, when it performed worse, the importance of salinity performed poorly. Temperature > depth > salinity was the resulting evaluation in order of decreasing relevance of each environmental component in determining the main population of hairtail.

It may be prejudicial to compare accuracy and importance separately, and there may be situations where one is sacrificed while the other is improved. For example, the winter depth factor provides greater coverage (up to 64%) while decreasing accuracy (Table 4, with an effective rate of only 28%). To avoid the above, we calculated an integrated fitness index ($I_{comp}$) for comparison using the following formula: $I_{comp}=\sqrt{R_{effectiv}\times R_{covering}}$. The results are presented in Table 7. The results of the average integrated suitability index for each season show that the order of excellence of the environmental factors is: temperature > salinity > depth.

The relative importance of temperature has been extensively studied in relation to the impacts of other environmental parameters on living beings. Temperature can affect how marine species are distributed across latitudes [31], and global warming conditions show a general trend towards there being fewer cold-water species and more warm-water species, as well as fewer polar species and more tropical species [31,32]. Marine animals can be classified into a variety of types based on their preferred water temperature [32,33]. Although salinity’s impacts on organisms are comparatively significant, many of them require temperature conditions to occur. Some currents, such as the Kuroshio, have high temperatures and high salinities, and their seasonal and interannual variations have a significant impact on the circulation pattern and the distribution of temperature and salinity in China’s coastal waters [34]. These currents also have special effects on the organisms they carry. Most research focuses on the combined influence of bathymetry and other factors, making the effect of bathymetry on organisms alone rather uncommon. To date, the effect of bathymetry on hard-bottom-fixed marine organisms has been the only single-factor analysis in the literature [35]. The combined effects of numerous elements are still the subjects of the greatest research. This paper compares the relative strengths of strong and weak effects of three environmental factors on the distribution of hairtail, which offers results essentially consistent with the findings of previous related studies and may also offer some guidance for the future related studies.

### 4.3. Seasonal Variations in Interactions between the Major Hairtail Population and Environmental Influences

According to this study, there are noticeable seasonal changes in coverage ratios that represent how important or useful environmental conditions are for finding the major hairtail populations. Compared to summer and winter, when coverage rates were greater than 50%, coverage of specific environmental factors was much lower in spring and autumn, with coverage ratios below 50% (Table 4). Similar characteristics were observed in the coverage that the composite factors were able to produce (Table 5); that is, the composite coverage in spring and autumn, whether created with two or three factors, was much lower than the coverage in summer and winter. The hairtail spatial distribution (Figure 2) also revealed changes in patterns between the four seasons, with spring and autumn tending towards offshore dispersal and summer and winter populations being primarily distributed in coastal waters.

The spring group and the autumn group are two different reproductive cycle groups for hairtail, according to the literature [36,37]. Due to their physiological needs, individuals in the reproductive cycle will travel to suitable spawning waters. According to studies conducted in these waterways [38,39], these waters have very particular environmental characteristics that can be used to stimulate or promote fish spawning. We can also explain the comparatively poor coverage in spring and autumn that was observed in this study. Differing environmental requirements between reproductive and other populations result in relatively scattered distributions among spawners and non-spawners. This increases the difficulty of achieving comprehensive coverage of all populations within the environmental range, consequently leading to a decrease in the coverage ratio. The distribution waters of the summer and winter groups, which primarily belong to the bait and overwintering groups, are very uniform and mostly spread along the inshore waters. As a result, the coverage of each environmental component increases significantly in both summer and winter.

### 4.4. Research Prospects

The research demonstrates that using increasing salinity and water depth as environmental factors to reduce non-target ranges as determined only by temperature in order to improve the accuracy of identifying the main range of the object is not successful in the case of hairtail. A more thorough comparison between these two water areas can be considered in the next step if we wish to continue investigating the reasons for the variations in the organism’s aggregation between different water areas under similarly favorable environmental conditions. The research findings of this study did not yield the desired outcomes, possibly due to the limited suitability of the selected environmental factors. Given this limitation, we recognize the importance of contemplating additional environmental candidates for inclusion in our research framework. Future investigations may explore factors such as predator distribution, chlorophyll blooming and ocean currents, which may contribute to a more comprehensive understanding of hairtail aggregation patterns.

The major populations within temperature distribution and water depth were quite continuous during the investigation, while the main salinity distribution was more dispersed. Additionally, the major salinity distribution in all seasons revealed one or more blank waters emanating from the center of the continuously dispersed waters, and these blank waters had the shapes of continuous circles or, in the cases of some, distorted polygons. This could be a result of the rapid emergence of groundwater and total separation from the environment, which led to a rapid decline in salinity. More research is needed to understand the exact mechanism of formation, and beneficial attempts and explorations can be made regarding the impact of such special environments on fisheries organisms.

## 5. Conclusions

Based on the original objectives of this study, the attempted approach did not produce successful results. However, it does provide a framework for investigating the influence of multiple environmental factors on the spatial distribution of organisms. The key is to identify the relevant environmental factors that effectively constrain the distribution ranges of organisms in order to obtain accurate spatial mapping. In future investigations, a comparative analysis between aggregated and non-aggregated water areas within a single factor can shed light on other determinants of organism distribution.

In the case of the hairtail, the priority order of the environmental factors that determine its spatial distribution is temperature > salinity > water depth. It was observed that the coverage of environmental factors had lower values in spring and autumn, and higher values in summer and winter. Moreover, hairtail population distributions tend to be closer to the coast during summer and winter, while being farther offshore during spring and autumn. All the above provide valuable information for future research on the spatial distribution and life-history of the hairtail.

## Figures and Tables

**Table 1:** Temperature-range related parameters of the primary hairtail at various seasons.

Season	Proportion	Distribution Type	Average	Temperature Range (°C)
Spring	1	norm	19.27 ± 0.60	18.67~19.87
Summer	1	norm	21.70 ± 2.03	19.67~23.72
Autumn	1	norm	20.34 ± 1.07	19.27~21.41
Winter	1	norm	15.95 ± 1.81	14.14~17.76

**Table 2:** Parameters related to primary hairtail salinity ranges at various seasons.

Season	Proportion	Distribution Type	Average	Salinity Range (‰)
Spring	1.00	norm	34.37 ± 0.11	34.26~34.49
Summer	0.24	norm	6.61 ± 0.34	6.28~6.95
	0.20	norm	30.92 ± 0.47	30.45~31.39
	0.32	norm	34.58 ± 0.37	34.21~34.95
Autumn	0.66	norm	32.21 ± 1.06	31.15~33.28
	0.25	norm	34.34 ± 0.12	34.22~34.46
Winter	0.07	norm	7.45 ± 0.80	6.65~8.25
	0.77	norm	33.94 ± 0.43	33.52~34.37

**Table 3:** Parameters related to the primary depth range for hairtail at various seasons.

Season	Proportion	Distribution Type	Average	Depth Range (m)
Spring	1.00	norm	71.86 ± 7.33	64.53~79.19
Summer	0.33	norm	38.83 ± 3.91	34.92~42.73
	0.66	norm	56.61 ± 8.79	47.82~65.4
	0.02	norm	96.62 ± 2.85	93.77~99.47
Autumn	0.76	norm	57.72 ± 9.41	48.31~67.13
	0.24	norm	95.02 ± 9.87	85.16~104.89
Winter	1.00	norm	74.43 ± 19.72	54.71~94.15

**Table 4:** Data statistics on the overlaps of spatial ranges between hairtail populations and environmental factors.

Item	Effective Area			Effective Ratio			Coverage Ratio		
	Temperature	Salinity	Depth	Temperature	Salinity	Depth	Temperature	Salinity	Depth
Spring	398	458	418	0.41	0.35	0.2	0.39	0.45	0.41
Summer	2164	1512	1913	0.52	0.38	0.39	0.71	0.5	0.63
Autumn	1845	1613	1690	0.42	0.3	0.44	0.45	0.39	0.41
Winter	1949	1542	1758	0.43	0.44	0.28	0.71	0.56	0.64
Average	1589	1281	1445	0.44	0.37	0.33	0.56	0.48	0.52
SD	805	550	691	0.05	0.06	0.11	0.17	0.07	0.13

**Table 5:** Data statistics on the overlaps of spatial ranges between hairtail populations and environmental multi-factor analyses.

Item	Temp. ∩ Sali.		Temp. ∩ Depth		Sali. ∩ Depth		Temp. ∩ Sali. ∩ Depth	
	Effective Area	Coverage Ratio	Effective Area	Coverage Ratio	Effective Area	Coverage Ratio	Effective Area	Coverage Ratio
Spring	228	0.22	283	0.28	301	0.30	170	0.17
Summer	1078	0.36	1478	0.49	998	0.33	797	0.26
Autumn	913	0.22	868	0.21	874	0.21	466	0.11
Winter	1353	0.49	1569	0.57	1281	0.46	1187	0.43
Average	893	0.32	1050	0.39	864	0.32	655	0.24
SD	479	0.13	598	0.17	412	0.10	437	0.14

**Table 6:** Statistical analysis of changes in coverage rate after the combination of environmental factors.

Item	Temp. ∩ Sali.		Temp. ∩ Depth		Sali. ∩ Depth		Temp. ∩ Sali. ∩ Depth		
	Temp.	Sali.	Temp.	Depth	Sali.	Depth	Temp. ∩ Sali.	Temp. ∩ Depth	Sali. ∩ Depth
Spring	−0.44	−0.51	−0.28	−0.32	−0.33	−0.27	−0.23	−0.39	−0.43
Summer	−0.49	−0.28	−0.31	−0.22	−0.34	−0.48	−0.28	−0.47	−0.21
Autumn	−0.51	−0.44	−0.53	−0.49	−0.46	−0.49	−0.5	−0.48	−0.48
Winter	−0.31	−0.13	−0.2	−0.11	−0.18	−0.28	−0.12	−0.25	−0.07
Average	−0.44	−0.34	−0.33	−0.28	−0.33	−0.38	−0.28	−0.4	−0.3
SD	0.09	0.17	0.14	0.16	0.11	0.12	0.16	0.11	0.19

**Table 7:** Composite fitness index of environmental factors.

Item	Temperature Composite Index	Salinity Composite Index	Depth Composite Index
Spring	0.40	0.40	0.29
Summer	0.61	0.44	0.50
Autumn	0.43	0.34	0.42
Winter	0.55	0.50	0.42
Average	0.50	0.42	0.41
SD	0.10	0.07	0.09

## Data Availability

The data presented in this study are available upon request from the corresponding author. The data are not publicly available due to a classification as confidential by a government agency, given an association with national security issues.
